# Endometrial scratch in women undergoing first-time IVF treatment: a systematic review and meta-analysis of randomized controlled trials

**DOI:** 10.1016/j.rbmo.2021.11.021

**Published:** 2022-04

**Authors:** Mostafa Metwally, Robin Chatters, David White, Jamie Hall, Stephen Walters

**Affiliations:** a Obstetrics, Gynaecology and Neonatology, Sheffield Teaching Hospitals NHS Foundation Trust and The University of Sheffield, Sheffield, UK; b Sheffield Clinical Trials Research Unit, School of Health and Related Research, The University of Sheffield, Sheffield, UK

**Keywords:** Endometrial scratch, First cycle, Induced endometrial trauma, IVF, Live birth rate, Systematic review

## Abstract

The endometrial scratch procedure is an IVF ‘add-on’ sometimes provided prior to the first IVF cycle. A 2019 systematic review concluded that there was insufficient evidence to show whether endometrial scratch has a significant effect on pregnancy outcomes (including live birth rate, LBR) when undertaken prior to the first IVF cycle. Further evidence was published following this review, including the Endometrial Scratch Trial (ISRCTN23800982). The objective of the current review was to synthesize and critically appraise the evidence for the clinical effectiveness and safety of the endometrial scratch procedure in women undergoing their first IVF cycle. Databases searched include MEDLINE, Embase, CINAHL and ClinicalTrials.gov. Eligible randomized controlled trials included women undergoing IVF for the first time that reported the effectiveness and/or safety of the endometrial scratch procedure; 12 studies were included. Meta-analysis showed no evidence of a significant effect of the endometrial scratch on LBR (10 trials, odds ratio [OR] 1.17, 95% confidence interval [CI] 0.76–1.79) or other pregnancy outcomes. This review confirms that there is a lack of evidence that endometrial scratch improves pregnancy outcomes, including LBR, for women undergoing their first IVF cycle. Clinicians are recommended not to perform this procedure in individuals undergoing their first cycle of IVF.

## Introduction

Endometrial scratch is a procedure that has been rapidly adopted into routine clinical practice at a rate that far exceeds the rate of production of good-quality evidence (Lensen *et al.*, 2016). While the procedure was initially adopted for women suffering from recurrent implantation failure during IVF treatment, based on evidence from the initial study by Barash *et al.* (2003), it then rapidly spread to other populations of women and other types of treatments.

Among these groups are women undergoing their first IVF cycle, where endometrial scratch had started to be offered despite the lack of evidence (Lensen *et al.*, 2016). Indeed, there have since been many studies, but these were mostly subject to significant confounding factors or not designed or powered to address this particular group (Vitagliano *et al.*, 2019).

A systematic review on this topic, focusing on randomized controlled trials (RCT) and the effectiveness of the endometrial scratch procedure in women undergoing their first IVF cycle, was published by Vitagliano *et al.* (2019). The review included seven RCTs and concluded that there was no evidence that the endometrial scratch followed by IVF compared with IVF alone increased the success of treatment, with a relative risk/risk ratio (RR) of live birth (or ongoing pregnancy if live birth rate [LBR] was not reported) of 0.99 (95% confidence interval [CI] 0.57–1.73, *P* = 0.97) (Vitagliano *et al.*, 2019). Secondary outcomes (miscarriage, multiple pregnancy and ectopic pregnancy) were also not significantly altered by undertaking the endometrial scratch. Notably, the small sample sizes of the included studies resulted in uncertainty around the effects of endometrial scratch in women undergoing their first IVF cycle, so a positive effect could not be ruled out. In addition, the trials included were at either a high or unclear risk of bias, making it difficult to draw reliable conclusions. Consequently, the authors concluded that a robust and definitive RCT is required to assess the effect of endometrial scratch on the chances of success of the first IVF cycle (Vitagliano *et al.*, 2019).

The current authors have recently published evidence from a large definitive multicentre RCT in the UK (the Endometrial Scratch Trial) that focused only on women undergoing their first IVF cycle, with or without intracytoplasmic sperm injection (ICSI) (Metwally *et al.*, 2021). This trial found no evidence for any significant benefit from the endometrial scratch. Given the large number of other studies in the literature, some with similar and some with conflicting findings, and given that often a meta-analysis of all published literature rather than a single RCT is important in propagating a certain research finding and implementing change in practice, the current meta-analysis was performed to synthesize the effect of endometrial scratch in increasing success rates of pregnancy outcomes in women undergoing first-time IVF treatment with or without ICSI.

## Materials and methods

The review was conducted, and this manuscript written, in accordance with the Preferred Reporting Items for Systematic Reviews and Meta-analysis (PRISMA) guidelines (Page *et al.*, 2021).

### Protocol registration

The systematic review was registered with PROSPERO (CRD42018111139, https://www.crd.york.ac.uk/PROSPERO/display_record.php?RecordID = 111139) on 18 October 2018.

### Study selection

Only RCTs examining the clinical effect or safety of endometrial scratch in women undergoing their first IVF cycle with or without ICSI, compared with treatment as usual (IVF/ICSI without the use of endometrial scratch), were eligible for inclusion. Studies that included participants undergoing intrauterine insemination (IUI) or ovulation induction (or other treatments not classed as IVF) and/or their second or subsequent IVF cycle, were excluded from this review, unless separate outcome data could be extracted for a subset of women who had undergone their first IVF cycle. All forms of endometrial scratch were included, regardless of the timing of the procedure during the cycle, but procedures defined as a mock transfer, where the aim of the procedure was not to scratch the endometrium but to test embryo transfer techniques, were excluded.

Reports published as abstracts only were excluded if insufficient methodological details were reported to allow extraction of study characteristics. Those published in languages other that English were also excluded, unless an English language abstract with sufficient methodological details existed.

### Outcome measures

The following clinical and safety outcome measures were considered, which were included regardless of the definition or timing of assessments: (i) primary: LBR; (ii) secondary: implantation rate, clinical pregnancy rate, ongoing pregnancy rate; miscarriage rate; ectopic pregnancy rate; pain related to the procedure; adverse and serious adverse event rates.

### Search methods for identification of studies

#### Data sources and search period

The following electronic databases were searched without language restrictions on 10 January 2020 (apart from ClinicalTrials.gov, which was searched on 21 September 2020): (i) MEDLINE via Ovid from 1948 to present (see Appendix in the **Supplementary Material**); (ii) Embase (Ovid) from 1980 to present; (iii) Cochrane Database of Systematic Reviews from 2005 to present; (iv) ClinicalTrials.gov (http://www.clinicaltrials.gov/); (v) Cumulative Index to Nursing and Allied Health Literature (CINAHL) from 1981 to present; (vi) CENTRAL via the Cochrane Register of Studies Online from 1898 to present.

Language restrictions were applied after the search was undertaken.

ClinicalTrials.gov was searched using combinations of keywords: ’endometrial biopsy’ and ‘infertility’, ‘endometrial biopsy’ and ‘subfertility’, ‘endometrial hysteroscopy’ and ‘infertility’, ‘endometrial hysteroscopy’ and ‘subfertility’.

The reference lists of all retrieved articles, relevant journals and conference proceedings were also searched by hand. In addition, authors were contacted to seek data clarification and to obtain additional information on missing data.

### Selection of studies and data extraction

Titles, abstracts and full-text articles were screened independently by two reviewers (JH and LR). Any disagreements regarding eligibility were resolved through discussion with RC.

Data were extracted from the studies by one researcher (JH) and all data checked by RC. Data extracted included the outcomes, study characteristics (e.g. country where research was conducted, number of trial arms, description of trial arms, control condition(s), timing of endometrial scratch procedure in menstrual cycle, device used for endometrial scratch) and participant characteristics (e.g. average age of trial population, average duration of infertility and egg source). Where further information was needed, the authors were contacted.

### Quality assessment strategy

The methodological quality of the included RCTs was assessed using the Cochrane Collaboration risk of bias assessment criteria at an outcome level (Sterne *et al.*, 2019). The risk of bias was assessed for each reported outcome. The assessment was undertaken independently by two reviewers (either PK, RC, AP or JH). Discrepancies were resolved by a third reviewer who had not been involved in the previous assessments of that study (RC or JH). Studies were graded with an overall risk of bias of ‘high’, ‘low’ or ‘unclear’.

### Data analysis

Statistical analysis was conducted according the guidelines outlined by the Cochrane Collaboration (Higgins *et al.*, 2021). For each included RCT, summaries on the number of events and the denominator were recorded for binary outcomes and meta-analysis performed using RevMan (Review Manager [RevMan]. Version 5.3, The Cochrane Collaboration, 2014). Study-specific treatment effects as measured by odds ratios (OR) and RR were combined to produce pooled OR or RR with 95% CI, where appropriate using the Mantel–Haenszel method, which performs relatively well in several settings (Piaget–Rossel and Taffé, 2019). A random-effects model was used when between-study heterogeneity was viewed as substantial; a fixed-effects model was used when there was no evidence of significant heterogeneity. Heterogeneity between studies was assessed using chi-squared and *I*^2^ statistics (Higgins and Thompson, 2002; Higgins *et al.*, 2003). For example, the *I*^2^ statistic quantifies the percentage of total variation in treatment effects estimates attributable to between-study heterogeneity; a value of >50% indicates evidence of significant heterogeneity of treatment effects between studies (Deeks *et al*., 2021). A subgroup analysis was conducted for two trials that reported ‘early’ miscarriages (prior to 12 weeks) (Izquierdo Rodriguez *et al*., 2020; Maged, 2018). Only one trial separately reported ‘late’ miscarriages (occurring between 12 and 24 weeks) (Izquierdo Rodriguez *et al*., 2020), with all other trials reporting miscarriages up to 24 weeks (which also included early miscarriages); therefore, due to the heterogeneity of this outcome, the late miscarriage subgroup was not included in the current analysis.

Where there was evidence of significant heterogeneity, a random-effects model was used in addition to an exploration of the causes of heterogeneity, followed by a sensitivity analysis where appropriate. Meta-analyses are presented in forest plots. Some outcomes (pain scores, adverse events) were narratively assessed due to a small number of studies reporting these outcomes, and/or heterogeneity in the definition of outcomes.

## Results

### Screening and study eligibility

**Figure 1:**
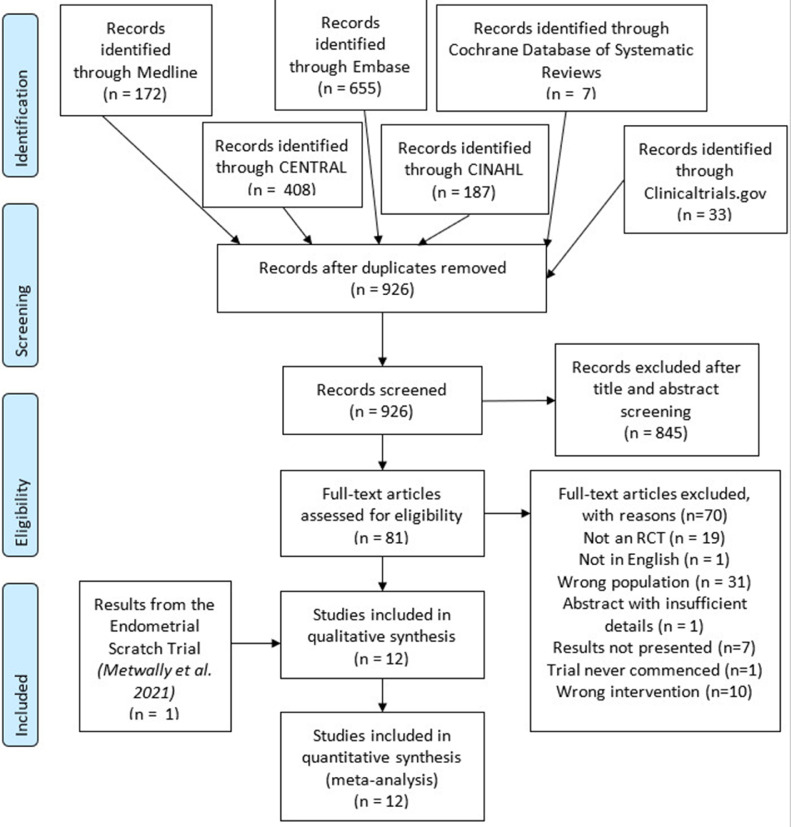
PRISMA flow chart. CENTRAL = Cochrane Register of Controlled Trials; CINAHL = Cumulative Index to Nursing and Allied Health Literature; RCT = randomized controlled trial.

Searches identified a total of 1462 records. When needed, authors were contacted regarding missing data and to help assess eligibility. Of the 14 authors that were contacted, eight were not available. One author confirmed that the trial was not eligible for inclusion as recruitment to the trial had not begun (Checa, 2013). The authors of four trials that included participants undergoing their first IVF cycle but did not present their outcomes separately provided data and were included in the review (Izquierdo Rodriguez *et al.*, 2020; Lensen *et al.*, 2019; Mackens *et al.*, 2020; Nastri *et al.*, 2013). Polanski *et al*. (2014), authors of a study that included women undergoing an unselected number of previous IVF cycles, could not be contacted to obtain data for first-cycle participants only. However, unpublished data received directly from the authors in a recent systematic review, were used (Vitagliano *et al.*, 2019). A risk of bias assessment could not be conducted for this study due to a lack of methodological details described in the abstract. After screening, 11 RCT were eligible for inclusion in the review (Eskew *et al.*, 2019; Izquierdo Rodriguez *et al.*, 2020; Karimzade *et al.*, 2010; Lensen *et al.*, 2019; Liu *et al.*, 2017; Mackens *et al.*, 2020; Maged *et al.*, 2018; Mahran *et al.*, 2016; Nastri *et al.*, 2013; Polanski *et al.*, 2014; Yeung *et al.*, 2014). Also included were the results of the recently conducted Endometrial Scratch Trial, published after the searches were undertaken, thus bringing the total to 12 RCTs (Metwally *et al.*, 2021) published between 2010 and 2021. Details of the literature search and study selection can be seen in Figure 1.

### Characteristics of included trials

Table 1 summarizes the characteristics of the 12 included RCT comprising 3382 participants undergoing their first IVF/ICSI cycle.

**Table 1:** Characteristics of included trials

Author (trial registration number)	Centre characteristics	Intervention / control	Inclusion criteria	No. of participants randomized undergoing first IVF cycle (total)	IVF/ICSI	Instrument used	Timing of ES	Outcomes
Karimzade *et al.*, 2010 (NCT00846183)	Single centre, Iran, June 2008 to January 2009	ES versus usual care	Age <38 years, BMI > 19 and <30 kg/m^2^, day 3 FSH < 12 mIU/ml Four to 14 oocytes retrieved	156	IVF with or without ICSI	Novak curette	During IVF Day of oocyte retrieval	CPR, OPR, IR, MAE
Yeung *et al.*, 2014 (NCT01977976)	Single centre, China, March 2011 to October 2013	ES versus usual care	Excluded donor eggs	209	IVF with or without ICSI	Pipelle	Prior to IVF 7 days post LH surge / day 21 of cycle preceding IVF	LBR, OPR, CPR, IR, MR, MPR, QP, MAE
Mahran *et al.*, 2016 (ISRCTN61316186)	Multicentre (two centres), Egypt, June 2012 to September 2014	ES versus usual care	20–40 years, FSH ≤12 mIU/ml and two or more good-quality embryos transferred	418	IVF with or without ICSI	Pipelle	Prior to IVF Day 21 to 24 of cycle preceding IVF	LBR, CPR, IR, MR, MPR, QP, MAE, EPR
Maged *et al.*, 2018 (NCT02660125)	Single centre, Egypt, January 2016 to March 2017	ES versus usual care	Age <40 years and FSH <10 mIU/ml	300	ICSI	Pipelle	Prior to IVF Luteal phase	CPR, IR, MR, MPR, MAE
Liu *et al.*, 2017 (ChiCTR-IOR-17011506)	Single centre, China, February 2012 to November 2014	ES (proliferative phase) / ES (luteal phase) versus sham procedure (proliferative phase) / sham procedure (luteal phase)	Age ≤40 years, FSH <12 mIU/ml	142	IVF with or without ICSI	Pipelle	Prior to IVF Proliferative phase (day 10 to 12) or luteal phase (day 7 to 9) of preceding cycle	LBR, CPR, IR, MR, EPR, MPR, QP, MAE
Eskew *et al.*, 2019 (Clinical trials registration unknown)	Single centre, USA, September 2013 to July 2017	ES versus usual care	Age 18–43 years	66	IVF	Pipelle	Prior to IVF 7–13 days post LH surge in cycle preceding IVF	LBR, CPR, MR
Lensen *et al.*, 2019 (ACTRN12614000 626662)	Multicentre (13 centres), New Zealand, Belgium, Sweden and UK	ES versus usual care	Age >18 years	626	IVF with or without ICSI	Pipelle	Prior to IVF Day 3 of the preceding cycle to day 3 of the IVF cycle	LBR, OPR, CPR, MR, EPR, MPR, SBR, NP, MAE, NAE
Mackens *et al.*, 2020 (NCT02061228)	Single centre, Belgium, April 2014 to October 2017	ES versus usual care	Age ≥18 and <40 years, BMI ≤35 or ≥18 kg/m^2^ and excluded donor eggs	148	IVF with or without ICSI	Pipelle	During IVF Days 6 to 7 of ovarian stimulation	LBR, CPR, MR, EPR, QP, MAE
Izquierdo Rodriguez *et al.*, 2020 (NCT03108157)	Single centre, Spain, January 2017 to October 2018	ES versus usual care	Only donor eggs	140	ICSI	Endometrial biopsy catheter	Prior to IVF Luteal phase: 5–10 days before the start of the period	LBR, CPR, OPR, IR, MR, MPR, EPR, NAE
Nastri *et al.*, 2013 (NCT01132144)	Single centre, Brazil, June 2010 to March 2012	ES versus sham procedure	Age <38 years	18	IVF with or without ICSI	Pipelle	Prior to IVF 7–14 days prior to planned start of ovarian stimulation	LBR, CPR, MR, NP, NAE
Metwally *et al.*, 2021 (ISRCTN23800982)	Multicentre (16 sites), UK, July 2016 to October 2018	Two arms, ES versus usual care	Age 18 to 37 years, BMI ≤35 kg/m^2^, FSH <10 mIU/ml	1048	IVF with or without ICSI	Pipelle	Prior to IVF Mid-luteal phase defined as 5–7 days before the expected next period, or 7–9 days after a positive ovulation test	LBR, CPR, IR, SBR, NP, MAE, PTR, MR, EPR, NAE
Polanski *et al.*, 2014 (NCT01882842)	Single centre, UK, February 2013 to June 2015	Two arms: ES, usual care	Age <49 years	111	IVF with or without ICSI	Pipelle or Wallace endometrial sampler	Prior to IVF 7–9 days post LH surge in the cycle preceding IVF	LBR, MPR, CPR

BMI = body mass index; CPR = clinical pregnancy rate; EPR = ectopic pregnancy rate; ES = endometrial scratch; ICSI = intracytoplasmic sperm injection; IR = implantation rate; LBR = live birth rate; MAE = maternal adverse events; MBR = multiple birth rate; MPR = multiple pregnancy rate; MR = miscarriage rate; NAE = neonatal adverse events; NP = numerical pain score; OPR = ongoing pregnancy rate; PTR = preterm delivery rate; QP = qualitative pain score; SBR = stillbirth rate.

### Nature of trials and geographical coverage

Only three of the 12 studies were multicentre RCTs (Lensen *et al.*, 2019; Mahran *et al.*, 2016; Metwally *et al.*, 2021), with other studies involving a single centre. All 12 studies were individually randomized and were conducted across ten countries: Iran, Egypt (two studies), China (two studies), USA, Belgium, Spain, Brazil and the UK (two studies). One RCT was undertaken multinationally across five countries (Lensen *et al.*, 2019). The total number of participants included in each trial who were undergoing their first IVF cycle ranged from 18 to 1048.

Eleven studies were two-arm RCTs (Eskew *et al.*, 2019; Izquierdo Rodriguez *et al.*, 2020; Karimzade *et al.*, 2010; Lensen *et al.*, 2019; Mackens *et al.*, 2020; Maged *et al.*, 2018; Mahran *et al.*, 2016; Metwally *et al.*, 2021; Nastri *et al.*, 2013; Polanski *et al.*, 2014; Yeung *et al.*, 2014) and one was a four-arm RCT (Liu *et al.*, 2017). Nine of the two-arm RCT compared the endometrial scratch procedure to usual care and two trials included a comparator involving a sham procedure (Liu *et al.*, 2017; Nastri *et al.*, 2013). The four-arm trial compared endometrial scratch at two different time points with a sham procedure undertaken at the same two different time points in the menstrual cycle – proliferative and luteal (Liu *et al.*, 2017).

Recruitment to three of the trials was prematurely ended due to an unplanned futility analysis showing no differences in clinical pregnancy rates between intervention and control groups in one trial (Eskew *et al.*, 2019), a planned interim analysis identifying higher miscarriage rates in the endometrial scratch arm in another trial (Mackens *et al.*, 2020), and identifying a significant benefit of the endometrial scratch during a planned interim analysis (Nastri *et al.*, 2013).

### Characterization of the endometrial scratch procedure and timing

The method of undertaking endometrial scratch was largely similar across studies. Most used a Pipelle sampler to invoke injury, except for one trial that used an embryo transfer catheter (Izquierdo Rodriguez *et al.*, 2020), one that used a Novak curette (Karimzade *et al.*, 2010), and another that used either a Pipelle or Wallace endometrial sampler (Polanski *et al.*, 2014). However, there was substantial variation in the timing of when endometrial scratch was performed across trials. Two trials undertook endometrial scratch during the IVF cycle, either on the day of egg collection (Karimzade *et al.*, 2010), or during ovarian stimulation (Mackens *et al.*, 2020). Ten trials undertook endometrial scratch in the menstrual cycle prior to IVF, with seven within the luteal phase (Eskew *et al.*, 2019; Izquierdo Rodriguez *et al.*, 2020; Maged *et al.*, 2018; Mahran *et al.*, 2016; Metwally *et al.*, 2021; Polanski *et al.*, 2014; Yeung *et al.*, 2014), and one during the early or mid-luteal phase (Nastri *et al.*, 2013). Lensen *et al.* (2019) undertook endometrial scratch at any point between day 3 of the menstrual cycle prior to endometrial scratch and day 3 of the cycle in which IVF was being undertaken. However, this trial reported that the median time (interquartile range, IQR) between endometrial scratch and embryo transfer was 35 days (22–39), and therefore it is likely that most women received endometrial scratch in the menstrual cycle prior to IVF. Liu *et al.* (2017) undertook endometrial scratch either in the proliferative or luteal phases of the menstrual cycle, therefore in this review the two time points of delivery of endometrial scratch, or the sham procedure, were combined, so that, for each outcome, there was one rate for the endometrial scratch arm (both proliferative and luteal), and another for the sham arm (both proliferative and luteal). Two trials provided hysteroscopy to all trial participants, prior to IVF (Mahran *et al.*, 2016; Yeung *et al.*, 2014).

### Participant eligibility

Trials used different participant eligibility criteria. Nine trials had age restrictions with an upper limit of between 35 and 49 years (Eskew *et al.*, 2019; Karimzade *et al.*, 2010; Liu *et al.*, 2017; Mackens *et al.*, 2020; Maged *et al.*, 2018; Mahran *et al.*, 2016; Metwally *et al.*, 2021; Nastri *et al.*, 2013; Polanski *et al.*, 2014). Three trials restricted the body mass index (BMI) to an upper limit ranging from 30 to 35 kg/m^2^ (Karimzade *et al.*, 2010; Mackens *et al.*, 2020; Metwally *et al.*, 2021). Five trials selected women that were deemed to have a good ovarian reserve, by allowing only those with a certain concentration of FSH to participate, with a maximum concentration of 10 IU/ml in two studies (Maged *et al*., 2018; Metwally *et al*., 2021) and 12 IU/ml in three studies (Karimzade *et al*., 2010; Liu *et al*., 2017; Mahran *et al*., 2016). At eligibility screening, two studies set requirements for the number of oocytes collected or embryos transferred: two or more embryos transferred in one study (Mahran *et al.*, 2016), and four to 14 oocytes collected in another (Karimzade *et al.*, 2010). Only one trial stipulated that the embryos transferred had to be of a certain quality, with Mahran *et al.* (2016) stating that the two or more embryos transferred had to be ‘good’ quality. However, the exact method of grading embryos or defining a good-quality embryo was not reported. Two trials excluded women receiving donor eggs (Mackens *et al.*, 2020; Yeung *et al.*, 2014), while one study only included those receiving donor eggs (Izquierdo Rodriguez *et al.*, 2020).

### Trial outcomes

Ten trials reported LBR (Eskew *et al.*, 2019; Izquierdo Rodriguez *et al.*, 2020; Lensen *et al.*, 2019; Liu *et al.*, 2017; Mackens *et al.*, 2020; Mahran *et al.*, 2016; Metwally *et al.*, 2021; Nastri *et al.*, 2013; Polanski *et al.*, 2014; Yeung *et al.*, 2014). Clinical pregnancy rates were reported in all trials. However, this was defined inconsistently, with marked variation in the time point at which this outcome was assessed: at 4 weeks post embryo transfer in two trials (Maged *et al.*, 2018; Mahran *et al.*, 2016); 5 weeks in one trial (Karimzade *et al.*, 2010); 6 weeks in four trials (Izquierdo Rodriguez *et al.*, 2020; Lensen *et al.*, 2019; Liu *et al.*, 2017; Yeung *et al.*, 2014); 7 weeks (Mackens *et al.*, 2020) and 8 weeks (Metwally *et al.*, 2021); and not defined in three trials (Eskew *et al.*, 2019; Nastri *et al.*, 2013; Polanski *et al.*, 2014). Ongoing pregnancy rates were reported in four trials, which were assessed at 12 weeks (Izquierdo Rodriguez *et al.*, 2020; Karimzade *et al.*, 2010; Lensen *et al.*, 2019) and 20 weeks (Yeung *et al.*, 2014) post embryo transfer.

Implantation rates were reported in seven studies (Izquierdo Rodriguez *et al.*, 2020; Karimzade *et al.*, 2010; Liu *et al.*, 2017; Maged *et al.*, 2018; Mahran *et al.*, 2016; Metwally *et al.*, 2021; Yeung *et al.*, 2014). Six studies defined this similarly as the number of gestational sacs divided by the number of embryos transferred, while in Metwally *et al.* (2021) this was defined as the number of gestational sacs divided by the number of participants randomized to each arm (under intention-to-treat principles). Therefore, in order to include the current trial in this meta-analysis, this outcome was recalculated using the number of embryos transferred as the denominator. Miscarriage rates per clinical pregnancy were reported in 11 trials (Eskew *et al.*, 2019; Izquierdo Rodriguez *et al.*, 2020; Lensen *et al.*, 2019; Liu *et al.*, 2017; Mackens *et al.*, 2020; Maged *et al.*, 2018; Mahran *et al.*, 2016; Metwally *et al.*, 2021; Nastri *et al.*, 2013; Polanski *et al.*, 2014; Yeung *et al.*, 2014), with the time point of data collection differing between 12 and 24 weeks of gestation, but unclear in two trials (Lensen *et al.*, 2019; Mackens *et al.*, 2020). A subjective assessment of pain of the endometrial scratch procedure on a numerical rating scale was reported in three trials (Lensen *et al.*, 2019; Metwally *et al.*, 2021; Nastri *et al.*, 2013), with four studies providing qualitative reports of pain (Liu *et al.*, 2017; Mackens *et al.*, 2020; Mahran *et al.*, 2016; Yeung *et al.*, 2014). Eight trials reported adverse events and/or complications in the participating women (Karimzade *et al.*, 2010; Lensen *et al.*, 2019; Liu *et al.*, 2017; Mackens *et al.*, 2020; Maged *et al.*, 2018; Mahran *et al.*, 2016; Metwally *et al.*, 2021; Yeung *et al.*, 2014), and four trials reported such events in the baby or neonate (Izquierdo Rodriguez *et al.*, 2020; Lensen *et al.*, 2019; Metwally *et al.*, 2021; Nastri *et al.*, 2013).

### Risk of bias assessment

**Figure 2:**
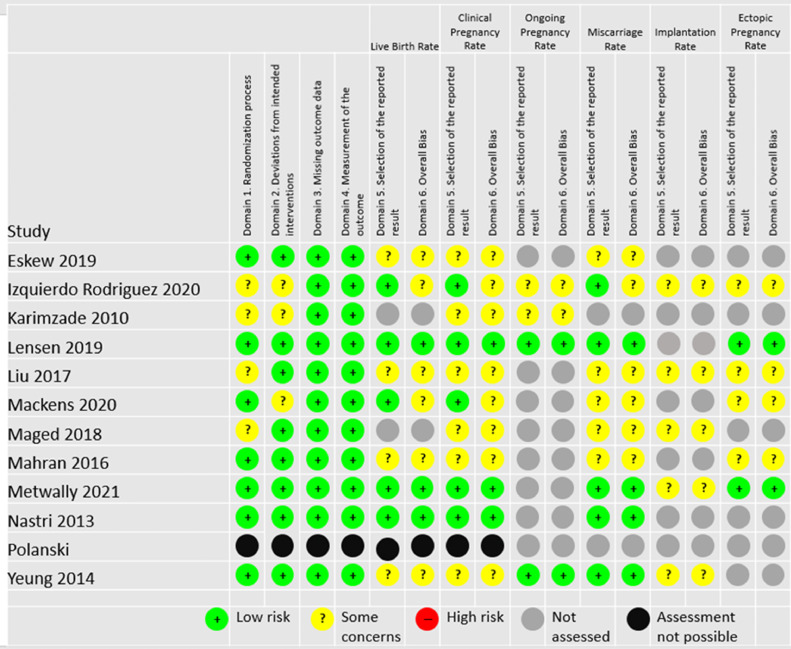
Risk of bias assessment.

It was not possible to conduct a risk of bias assessment for Polanski *et al.* (2014) as the author did not respond to a request for essential missing information. The only information available was from a recent review, which used a previous version of the risk of bias tool and therefore the authors’ assessments could not be considered in this review (Polanski *et al.*, 2014). For other included studies, Figure 2 summarizes the assessment of the risk of bias.

Domains 1 to 3 were consistent across all outcomes per study. Four trials were assessed as ‘some concerns’ in Domain 1 (allocation concealment) (Izquierdo Rodriguez *et al.*, 2020; Karimzade *et al.*, 2010; Liu *et al.*, 2017; Maged *et al.*, 2018). Nine trials used a computerized system to undertake randomization (Eskew *et al.*, 2019; Izquierdo Rodriguez *et al.*, 2020; Karimzade *et al.*, 2010; Lensen *et al.*, 2019; Mackens *et al.*, 2020; Maged *et al.*, 2018; Metwally *et al.*, 2021; Nastri *et al.*, 2013; Yeung *et al.*, 2014), one trial used sealed envelopes (Mahran *et al.*, 2016), while another used a table of random numbers (Liu *et al.*, 2017).

Domain 2 considered the risk of bias due to deviations from the intended interventions. Three trials were considered to have some concerns of bias for this domain (Izquierdo Rodriguez *et al.*, 2020; Karimzade *et al.*, 2010; Mackens *et al.*, 2020). The differing dropout rate between the endometrial scratch group (8.5%) and the control group (2.2%) in Izquierdo Rodriguez *et al.* (2020) resulted in this assessment. Similarly, in Karimzade *et al.* (2010), there were four patients excluded from the analysis in the endometrial scratch arm only.

Domain 3 considered the risk of bias due to missing outcome data. The rate of missing data was low across all trials, therefore all were considered to be at low risk of bias for this domain.

All studies were judged to be at low risk of bias for Domain 4, ‘measurement of the outcome’.

Given the nature of the outcomes being assessed, patient knowledge of the intervention was unlikely to have affected the analysis. Therefore, even though only three of the 12 included studies involved some form of blinding (Eskew *et al.*, 2019; Liu *et al.*, 2017; Nastri *et al.*, 2013), this is not considered to affect the patient outcomes. There was also no blinding in most of the included trials, however it is unlikely that a participant's or clinician's knowledge of the intervention could have biased outcome assessment due to the included trials using objective outcome measures unlikely to be influenced by placebo effect.

Domain 5 assessed the risk of bias in selection of the reported result. For all outcomes, studies where the outcome was not specified prior to the start of the trial (no protocol, trials registry, or a retrospectively added trials registry), the timing of the outcome was not specified, or the outcome specification in the paper did not match the protocol/registry, were considered to have some concerns.

Only Lensen *et al.* (2019) and Nastri *et al.* (2013) were considered to have a low risk of bias across all assessments. Metwally *et al.* (2021) was denoted to have ‘some concerns’ for the implantation rate outcome only, as this was originally reported under intention-to-treat principles using the number of randomized women as the denominator in each arm; this was recalculated using the number of embryos transferred for the purposes of this meta-analysis (Metwally *et al.*, 2021). All other studies had at least one outcome considered to have some bias concerns.

### Live birth rate (LBR)

**Figure 3:**
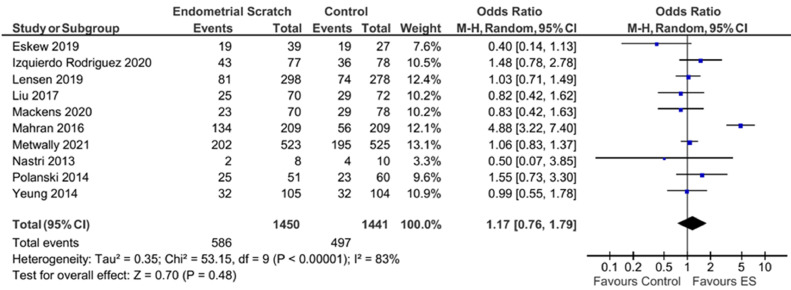
Forest plot showing the effect of endometrial scratch on live birth rate. CI = confidence interval; df = degrees of freedom; ES = endometrial scratch; M–H = Mantel–Haenszel.

Pooled analysis of the ten trials that reported LBR showed no evidence for a significant effect for endometrial scratch on the LBR (Figure 3, OR 1.17; 95% CI 0.76–1.79; *P* = 0.48) (Eskew *et al.*, 2019; Izquierdo Rodriguez *et al.*, 2020; Lensen *et al.*, 2019; Liu *et al.*, 2017; Mackens *et al.*, 2020; Mahran *et al.*, 2016; Metwally *et al.*, 2021; Nastri *et al.*, 2013; Polanski *et al.*, 2014; Yeung *et al.*, 2014). There was a substantial between-study heterogeneity in treatment effects (*I*^2^ = 83%).

### Clinical pregnancy rate (CPR)

**Figure 4:**
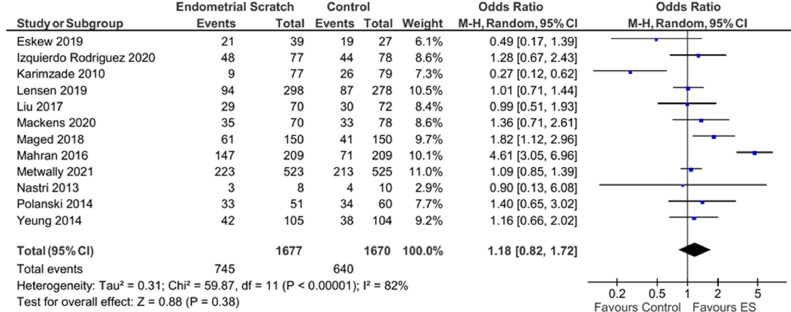
Forest plot showing the effect of endometrial scratch on clinical pregnancy rate. CI = confidence interval; df = degrees of freedom; ES = endometrial scratch; M–H = Mantel–Haenszel.

Pooled analysis of the 12 trials that reported CPR showed no evidence for a significant effect for endometrial scratch on the CPR (Figure 4, OR 1.18; 95% CI 0.82–1.72; *P* = 0.38) (Eskew *et al.*, 2019; Izquierdo Rodriguez *et al.*, 2020; Karimzade *et al.*, 2010; Lensen *et al.*, 2019; Liu *et al.*, 2017; Mackens *et al.*, 2020; Maged *et al.*, 2018; Metwally *et al.*, 2021; Nastri *et al.*, 2013; Polanski *et al.*, 2014; Yeung *et al.*, 2014). Results showed evidence for significant heterogeneity between the included studies (*I*^2^ = 82%).

### Ongoing pregnancy rate (OPR)

**Figure 5:**
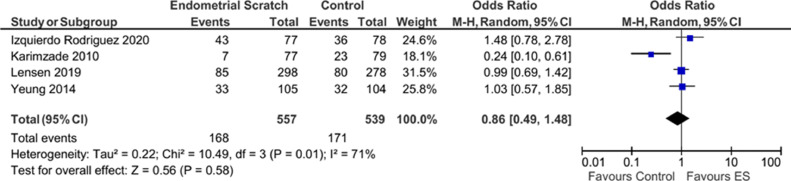
Forest plot showing the effect of endometrial scratch on ongoing pregnancy rate. CI = confidence interval; df = degrees of freedom; ES = endometrial scratch; M–H = Mantel–Haenszel.

Pooled analysis of the four studies that reported on OPR (Izquierdo Rodriguez *et al.*, 2020; Karimzade *et al.*, 2010; Lensen *et al.*, 2019; Yeung *et al.*, 2014) showed no evidence for a significant effect for endometrial scratch on the OPR (Figure 5, OR 0.86; 95% CI 0.49–1.48; *P* = 0.58). Results showed evidence for significant heterogeneity between the included studies (*I*^2^ = 71%).

### Implantation rate

**Figure 6:**
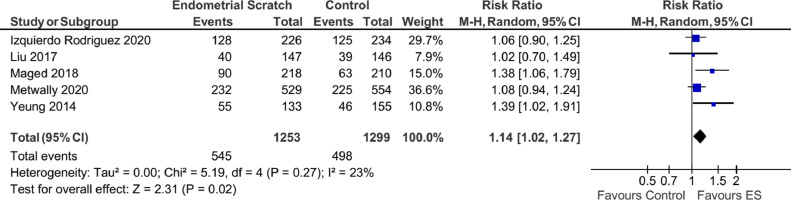
Forest plot showing the effect of endometrial scratch on implantation rate. CI = confidence interval; df = degrees of freedom; ES = endometrial scratch; M–H = Mantel–Haenszel.

Two trials (Karimzade *et al.*, 2010; Mahran *et al.*, 2016) where the implantation rate was only reported as a percentage without the absolute numbers were excluded from this analysis. Consequently, five trials (Izquierdo Rodriguez *et al.*, 2020; Liu *et al.*, 2017; Maged *et al.*, 2018; Metwally *et al.*, 2021; Yeung *et al.*, 2014) were included and the overall effect showed evidence to support a significant improvement in the implantation rate attributed to the use of endometrial scratch (Figure 6, OR 1.14; 95% CI 1.02–1.27; *P* = 0.02), with moderate evidence of heterogeneity (*I*^2^ = 23%).

### Miscarriage rate

**Figure 7:**
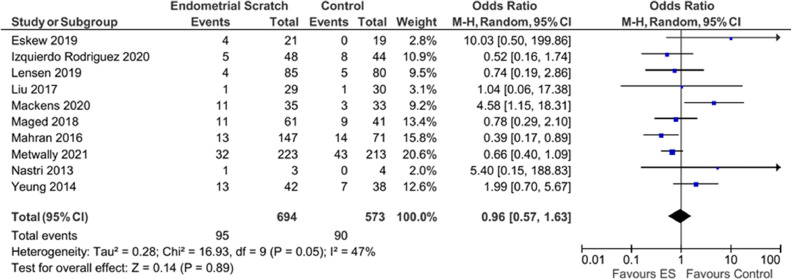
Forest plot showing the effect of endometrial scratch on miscarriage rate. CI = confidence interval; df = degrees of freedom; ES = endometrial scratch; M–H = Mantel–Haenszel.

Analysis of ten trials reporting on this outcome (Eskew *et al.*, 2019; Izquierdo Rodriguez *et al.*, 2020; Lensen *et al.*, 2019; Liu *et al.*, 2017; Mackens *et al.*, 2020; Maged *et al.*, 2018; Mahran *et al.*, 2016; Metwally *et al.*, 2021; Nastri *et al.*, 2013; Yeung *et al.*, 2014) did not show evidence for a significant effect of endometrial scratch on reducing the miscarriage rate (Figure 7, OR 0.96; 95% CI 0.57–1.63; *P* = 0.89). Results showed evidence for moderately significant heterogeneity (*I*^2^ = 47%).

### Subgroup analysis

**Figure 8:**
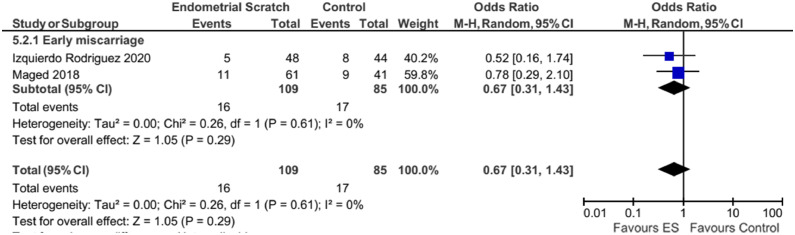
Forest plot showing the effect of endometrial scratch on early miscarriage rate. CI = confidence interval; df = degrees of freedom; ES = endometrial scratch; M–H = Mantel–Haenszel.

A subgroup analysis was performed on ‘early’ miscarriages (before 12 weeks), which was reported by two studies (Izquierdo Rodriguez *et al.*, 2020; Maged, 2018). Results remained unchanged as there was no evidence for a significant effect for endometrial scratch on the early miscarriage rate, with no evidence of significant heterogeneity between the included studies (*I*^2^ = 0%), but with significant uncertainty in this result (Figure 8, early: OR 0.67; 95% CI 0.31–1.43; *P* = 0.29).

### Ectopic pregnancy rate (EPR)

**Figure 9:**
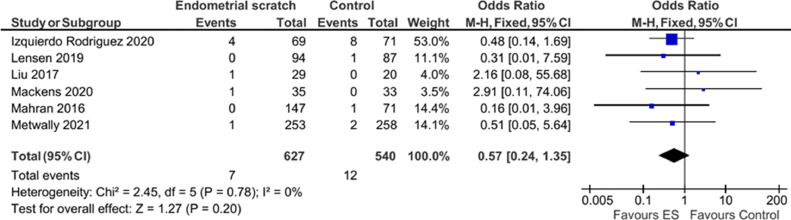
Forest plot showing the effect of endometrial scratch on ectopic pregnancy rate. CI = confidence interval; df = degrees of freedom; ES = endometrial scratch; M–H = Mantel–Haenszel.

Pooled analysis of the six studies that reported ectopic pregnancy (Izquierdo Rodriguez *et al.*, 2020; Lensen *et al.*, 2019; Liu *et al.*, 2017; Mackens *et al.*, 2020; Mahran *et al.*, 2016; Metwally *et al.*, 2021), showed no evidence for a significant effect for endometrial scratch on this outcome, with high uncertainty (Figure 9, OR 0.57; 95% CI 0.24–1.35; *P* = 0.20). There was no evidence of significant heterogeneity between studies (*I*^2^ = 0%), but there was high uncertainty in this estimate.

### Pain

It was not possible to perform a meta-analysis on the effect of endometrial scratch procedure on the pain experienced by participants as only three trials reported a quantitative outcome used to assess pain (Lensen *et al.*, 2019; Metwally *et al.*, 2021; Nastri *et al.*, 2013). Two of the trials reported similar pain levels (on a rating scale of 0 to 10, mean ± SD) directly post procedure (4.1 ± 2.4) and during the procedure (4.2 ± 2.5) (Lensen *et al.*, 2019; Metwally *et al.*, 2021), whereas the third reported slightly higher pain levels (6.42 ± 2.35) directly after the procedure (Nastri *et al.*, 2013). Other trials reported pain qualitatively, with four trials reporting a low proportion of participants that reported severe pain post procedure (0/150 in Yeung *et al.* (2014), 3/209 (1.4%) in Mahran *et al*. (2016), 0/70 in Liu *et al.* (2017), 2/70 (2.9%) in Mackens *et al*. (2020)).

### Adverse events

#### Maternal

Of the eight trials that reported adverse events, seven appeared to have collected adverse events in the endometrial scratch arm only, although information was often not presented in the reports. Four trials reported that there were ‘no complications’ recorded (Karimzade *et al.*, 2010; Liu *et al.*, 2017; Maged *et al.*, 2018; Yeung *et al.*, 2014) and one trial reported ‘minimal’ spotting for a few days after endometrial scratch (number of participants not reported) (Mahran *et al.*, 2016).

One trial reported that four participants (1.3%) experienced fainting, two (0.7%) reported excessive pain and one (0.3%) reported excessive bleeding (Lensen *et al.*, 2019). Another trial reported that three participants (5%) experienced bleeding (Mackens *et al.*, 2020). Metwally *et al.* (2021) was the only study to report safety events in both trial arms, and found comparable/similar incidences of adverse events between groups.

#### Neonatal

Four trials reported adverse events in the baby or neonate (Izquierdo Rodriguez *et al.*, 2020; Lensen *et al.*, 2019; Metwally *et al.*, 2021; Nastri *et al.*, 2013). The incidences of adverse events in participants undergoing the first or subsequent cycle of IVF in these trials was low, with no ‘major’ fetal malformations reported in one trial (Nastri *et al.*, 2013), rare congenital abnormalities (endometrial scratch arm: 3 [1.6%], treatment as usual [TAU] arm: 0 [0%]), fetal growth restriction (endometrial scratch: 10 [5.2%], TAU: 8 [4.3%]) and neonatal death (endometrial scratch: 0 [0%], TAU: 1 [0.5%]) in another trial (Lensen *et al.*, 2019) and rare placentation abnormalities (endometrial scratch: 1 [1.0%], TAU: 0 [0%]) and intrauterine growth restriction (endometrial scratch: 0 [0%], TAU: 1 [1%]) in the final trial (Izquierdo Rodriguez *et al.*, 2020). Metwally *et al.* (2021) was the only trial to present accessible data separately for those babies born to participants undergoing their first IVF cycle, reporting no neonatal deaths in both arms, no severe congenital abnormalities in the endometrial scratch arm and low numbers of congenital abnormalities in both trial arms.

### Sensitivity analysis

A sensitivity analysis was performed after exclusion of studies that were thought to be contributing to the heterogeneity, to explore the impact on the conclusions. Four such studies were identified for exclusion from the analyses (Karimzade *et al.*, 2010; Mackens *et al.*, 2020; Mahran *et al.*, 2016; Polanski *et al.*, 2014).

Mahran *et al.* (2016) appeared to be a clear outlier both statistically and methodologically. Firstly, this was the only trial to require participants to have two good-quality embryos transferred to be eligible to participate; including such a stipulation prior to randomization may have resulted in a participant population not comparable to other trials included in this review. Secondly, all participants received hysteroscopy prior to IVF, a procedure which in theory could have an effect similar to endometrial scratch, thus exposing the endometrium to two rather than one event of controlled endometrial trauma. Finally, an average of three embryos were transferred, while all other trials included in this review averaged one or two embryos transferred.

Polanski *et al.* (2014) included women who had undergone an unselected number of previous IVF cycles and could not be contacted to obtain data for first-cycle participants only. However, unpublished data pertaining to the participants that had undergone their first IVF cycle presented in a recent systematic review were used (Vitagliano *et al.*, 2019). A risk of bias assessment therefore could not be conducted for this study due to a lack of methodological details described in the abstract. Mackens *et al.* (2020) and Karemizade *et al.* (2010) were the only studies to undertake endometrial scratch during the IVF cycle, while the other trials undertook endometrial scratch in the menstrual cycle prior to endometrial scratch.

In summary, exclusion of these four studies eliminated or resulted in significant reduction in heterogeneity, but conclusions on the effect of endometrial scratch on LBR (**Supplementary Figure 1**, OR 1.01; 95% CI 0.91–1.31), CPR (**Supplementary Figure 2**, OR 1.12; 95% CI 0.96–1.32), OPR (**Supplementary Figure 3**, OR 1.08; 95% CI 0.82–1.42, miscarriage rate (**Supplementary Figure 4**, OR 0.87; 95% CI 0.62–1.22) and EPR (**Supplementary Figure 5**, OR 0.56; 95% CI 0.23–1.42) remained consistent and unchanged.

## Discussion

This systematic review and meta-analysis of RCTs in women undergoing the endometrial scratch procedure prior to their first cycle of IVF has identified that there is no evidence of a significant effect on LBR, CPR, OPR, EPR or miscarriage rate. These conclusions were consistent following sensitivity analyses excluding four studies that were contributing to significant heterogeneity. Uncertainty was high for some analyses, specifically miscarriage and ectopic pregnancy rates. A meta-analysis of implantation rates across five studies showed a significant positive effect of undertaking endometrial scratch. However, due to the sensitivity of implantation rate to the number of embryos transferred, and artificial inflation of a sample size when more than one embryo is transferred per woman, these results are unreliable and should be interpreted with extreme caution (Griesinger, 2016). Only six trials reported ectopic pregnancy rates, and for those trials that did, there were very few events reported, and surprisingly smaller trials reported more events than larger trials. This is difficult to explain, but may be attributable to trials using different definitions for this outcome. Seven trials reported pain post endometrial scratch; four of these trials reported a low proportion of participants experiencing ‘severe’ pain post endometrial scratch. Three trials reported similar moderate post endometrial scratch pain ratings. The procedure appears to be safe, with studies reporting very rare complications post endometrial scratch. None of the included trials were deemed to be at a high risk of bias.

Numerous systematic reviews have been undertaken to assess the clinical effectiveness of the endometrial scratch procedure in the first IVF cycle. However, previous reviews have been unable to conclude the effect of endometrial scratch on the first cycle of IVF, due to a lack of definitive, high-quality evidence (Nastri *et al*., 2012; Potdar *et al*., 2012; Vitagliano *et al*., 2019). Following the completion of the Endometrial Scratch Trial, the results of which are included in this review, it can now be concluded that endometrial scratch should not be undertaken prior to the first cycle of IVF.

The ease and simplicity of the endometrial scratch procedure has perhaps been the main reason for its rapid adoption into routine clinical practice, initially without the support of good-quality research. The main problem that has afflicted previous studies is the inclusion of different and heterogeneous populations of participants and clinical practices. This has led to heterogeneity and a lack of reliability of evidence when addressing any one specific population. The current study focuses only on the population of women undergoing their first IVF cycle, with or without ICSI.

There have been several key studies published over the last 7 years that are relevant to women undergoing first-time IVF treatment. These include the studies by Yeung *et al*. (2014), Lensen *et al.* (2019) and the most recent study from this author group (Metwally *et al.*, 2021). The first of these studies (Yeung *et al*., 2014) was conducted in an unselected population of women undergoing IVF, of whom nearly 70% were having their first IVF cycle and subgroup analysis of this group (*n* = 209 of *n* = 300 individuals included in this trial) similarly found no difference in OPR between groups (Yeung *et al.*, 2014). However, in this study a mixture of protocols was used and there were no restrictions regarding age or day of embryo transfer, with most patients receiving two embryo transfers. Similarly, the study by Lensen *et al*. (2019) combined a mixture of patients with different prognostic potential, with two main subgroups, the first being women with recurrent implantation failure and the second women who had had a maximum of one previous cycle. The latter group, although providing some useful reflections regarding women having endometrial scratch in the absence of recurrent implantation failure, cannot be used as a substitute for a well-designed study powered to just the initial IVF cycle. This was the aim in a recently published study (Metwally *et al.*, 2021), which was the first large multicentre RCT that was powered only to a population of potential good responders due to undergo their first IVF cycle, and accounted for many of the sources of heterogeneity that may have affected previous studies. Results showed that in this particular population, endometrial scratch had no clinical benefit although it was tolerable and safe. However, it cannot be ignored that the ease of use of endometrial scratch combined with the promises made by results of other studies regarding the ability of this procedure to revolutionize success rates, coupled with the numerous plausible physiological hypotheses that have been put forward regarding how this procedure may act to improve implantation, present a barrier to changing the current pattern of practice. This meta-analysis, which combines the study by the current authors with all relevant previous studies, helps to conclusively settle the debate regarding the use of this procedure in the first cycle of IVF, and at the same time it identifies the main problems with the previous literature, which are highlighted in a systematic and robust way.

The main strength of this study is identifying the largest sources of bias and heterogeneity in the literature. This was particularly useful when examining outcomes that were associated with a significant level of statistical heterogeneity. Consequently, the analysis was repeated after excluding the main studies that were identified as potential sources of heterogeneity (Karimzade *et al.*, 2010; Mackens *et al.*, 2020; Mahran *et al.*, 2016; Polanski *et al.*, 2014). This approach successfully identified the studies that were causing marked heterogeneity in treatment effects across studies, as shown by the significant decrease in statistical markers of heterogeneity after exclusion of these studies. However, the sensitivity analysis did not alter the overall findings and there remained no evidence for a significant improvement in fertility outcomes with the use of endometrial scratch.

Some outcomes were difficult to assess, in particular ectopic pregnancy rates, where half of the studies reported this outcome, with smaller trials reporting more events. Pain scores were also difficult to assess; those that did report this outcome did so differently. Adverse events were often not recorded in the control arms of studies, limiting the comparisons that can be made to ‘usual’ IVF treatment. Due to time constraints, it was not possible to contact all authors to collect information to aid assessment of the risk of bias. For one trial included in the review (Polanski *et al.*, 2014), it was not possible to obtain the full-text article or correspond with the author in order to obtain data, and therefore, data for this trial were extracted from a recent systematic review (Vitagliano *et al.*, 2019). As a result, a risk of bias assessment for this trial could not be undertaken. Key outcomes (e.g. miscarriage rate) were variably defined across the included trials; consensus regarding the definition of key fertility outcomes should be reached in order to ensure the results of future trials can be combined within meta-analyses.

The findings of this systematic review and meta-analysis conclusively confirm that there is no evidence that induced endometrial trauma improves IVF outcomes, including live birth and pregnancy rates, for women undergoing their first IVF cycle. It is therefore recommended that the endometrial scratch procedure is not undertaken in this population of women undergoing their first IVF cycle. Despite uncertainty over the effect of endometrial scratch on miscarriage and ectopic pregnancy outcomes, it is recommended that further research is not undertaken, due to endometrial scratch not having a significant effect on LBR.

## Appendix Supplementary materials

Image, application 1
