# Supplementary material for: Endometrial scratch in women undergoing first-time IVF treatment: a systematic review and meta-analysis of randomized controlled trials
Source: Reprod Biomed Online. 2022 Apr;44(4):617–29. doi: 10.1016/j.rbmo.2021.11.021 (PMC9089309; doi:10.1016/j.rbmo.2021.11.021)
Supplement: Supplementary file 1 [file mmc1.docx]

**Supplementary Figure 1 – Live birth rate sensitivity analysis**

CI – confidence interval, df – degrees of freedom, ES – Endometrial Scratch, M-H - Mantel-Haenszel


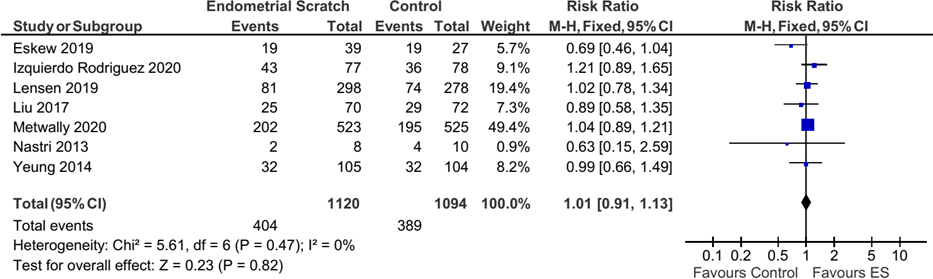


**Supplementary Figure 2 – Clinical pregnancy rate sensitivity analysis**

CI – confidence interval, df – degrees of freedom, ES – Endometrial Scratch, M-H - Mantel-Haenszel


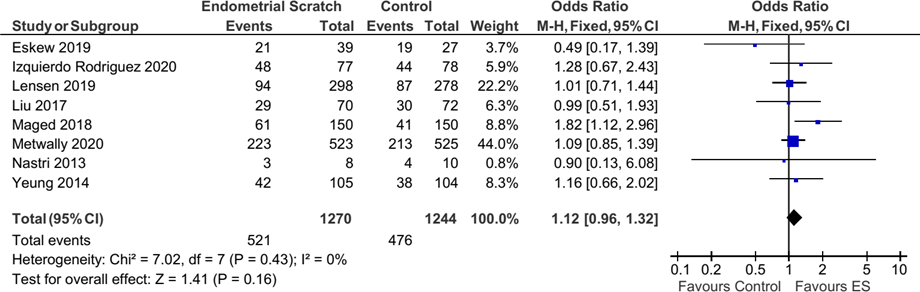


**Supplementary Figure 3 – Ongoing pregnancy rate sensitivity analysis**

CI – confidence interval, df – degrees of freedom, ES – Endometrial Scratch, M-H - Mantel-Haenszel

**
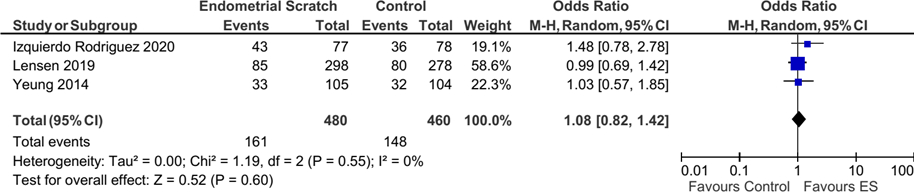
**

**Supplementary Figure 4 - Miscarriage rate sensitivity analysis**

CI – confidence interval, df – degrees of freedom, ES – Endometrial Scratch, M-H - Mantel-Haenszel

**
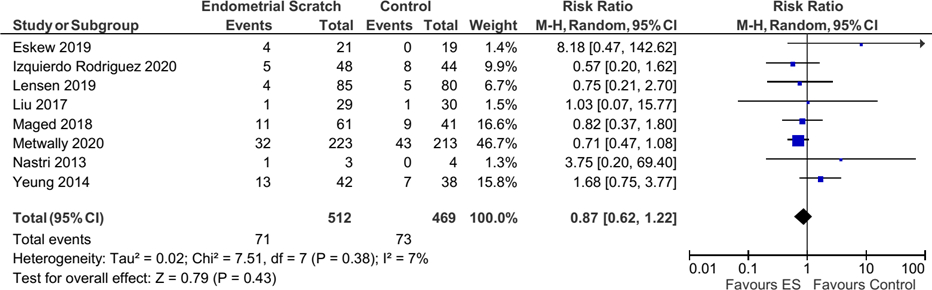
**

**Supplementary Figure 5– Ectopic pregnancy rate sensitivity analysis**

CI – confidence interval, df – degrees of freedom, ES – Endometrial Scratch, M-H - Mantel-Haenszel

**
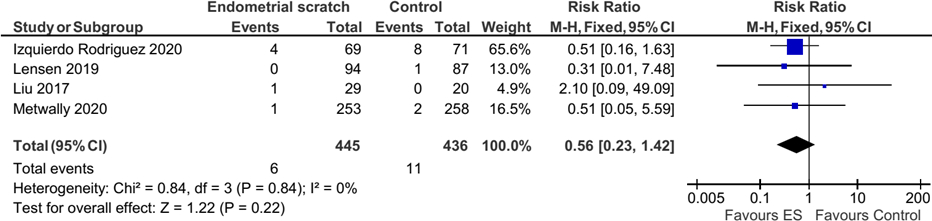
**
